# Identification of predictive models including polymorphisms in cytokines genes and clinical variables associated with post-transplant complications after identical HLA-allogeneic stem cell transplantation

**DOI:** 10.3389/fimmu.2024.1396284

**Published:** 2024-08-23

**Authors:** Paula Muñiz, María Martínez-García, Rebeca Bailén, María Chicano, Gillen Oarbeascoa, Juan Carlos Triviño, Ismael de la Iglesia-San Sebastian, Sara Fernández de Córdoba, Javier Anguita, Mi Kwon, José Luis Díez-Martín, Pablo M. Olmos, Carolina Martínez-Laperche, Ismael Buño

**Affiliations:** ^1^ Department of Hematology, Gregorio Marañón General University Hospital, Madrid, Spain; ^2^ Signal Theory and Communications Department, School of Engineering, Carlos III University, Leganés, Madrid, Spain; ^3^ Department of Signal Theory and Communications, University Carlos III of Madrid, Madrid, Spain; ^4^ Bioinformatic Department, Sistemas Genómicos, Valencia, Spain; ^5^ Department of Medicine, School of Medicine, Complutense University of Madrid, Madrid, Spain; ^6^ Genomics Unit, Gregorio Marañón General University Hospital, IiSGM, Madrid, Spain; ^7^ Department of Cell Biology, School of Medicine, Complutense University of Madrid, Madrid, Spain

**Keywords:** polymorphisms, graft-versus-host-disease, predictive models, cytokines, allogeneic transplantation

## Abstract

**Backgrounds:**

Although allogeneic hematopoietic stem cell transplantation (allo-HSCT) is a potentially curative therapy for hematological malignancies, it can be associated with relevant post-transplant complications. Several reports have shown that polymorphisms in immune system genes are correlated with the development of post-transplant complications. Within this context, this work focuses on identifying novel polymorphisms in cytokine genes and developing predictive models to anticipate the risk of developing graft-versus-host disease (GVHD), transplantation-related mortality (TRM), relapse and overall survival (OS).

**Methods:**

Our group developed a 132-cytokine gene panel which was tested in 90 patients who underwent an HLA-identical sibling-donor allo-HSCT. Bayesian logistic regression (BLR) models were used to select the most relevant variables. Based on the cut-off points selected for each model, patients were classified as being at high or low-risk for each of the post-transplant complications (aGVHD II-IV, aGVHD III-IV, cGVHD, mod-sev cGVHD, TRM, relapse and OS).

**Results:**

A total of 737 polymorphisms were selected from the custom panel genes. Of these, 41 polymorphisms were included in the predictive models in 30 cytokine genes were selected (17 interleukins and 13 chemokines). Of these polymorphisms, 5 (12.2%) were located in coding regions, and 36 (87.8%) in non-coding regions. All models had a statistical significance of p<0.0001.

**Conclusion:**

Overall, genomic polymorphisms in cytokine genes make it possible to anticipate the development all complications studied following allo-HSCT and, consequently, to optimize the clinical management of patients.

## 1 Introduction

Although allogeneic hematopoietic stem-cell transplantation (allo-HSCT) is a curative therapeutic approach for patients with hematologic malignancies, procedure-related morbidity and mortality may increase in the months or years following the procedure. In addition to the risk of malignancy relapse (30–40%), multiple factors such as drug-induced organ toxicity, infections and graft-*versus*-host disease (GVHD) compromise the full curative potential of allo-HSCT. Despite complications and mortality associated with transplantation have decreased in recent years, transplantation-related mortality (TRM) continues to be a major barrier to allo-HSCT. Several studies have found that 60–80% of TRM occurs within 100 days of transplantation. The increased use of reduced-intensity conditioning regimens and improvements in supportive care have reduced TRM.

Donor T cells promote hematopoietic engraftment, reconstitute T-cell immunity and mediate a potent beneficial antitumor effect known as graft-versus-leukemia (GVL) (1). Unfortunately, donor T cells also cause GVHD. GVHD occurs when the donor T cells within the graft identify the patient’s (host) healthy cells from various tissues as foreign, and attack and damage them (2). GVHD remains one of the major causes of morbidity following allo-HSCT, leading to prolonged use of immunosuppressive agents, organ dysfunction, increased risk of infection, and ultimately increased mortality (3). The manifestations and severity of GVHD are highly variable and are influenced by the proportions of naive cells maturing along regulatory T-cell, Th1, Th2, or Th17 phenotypes. This maturation is largely influenced by local cytokines, which, in turn, activate transcription factors and drive development toward a dominant phenotype. In addition, proinflammatory cytokines exert direct effects on GVHD target tissues. Genetic differences in non-HLA genes between recipients and donors are important, and the role of polymorphisms in cytokines and other immune related genes must be taken into account (4).

Although there are several studies that associate the presence of polymorphisms in cytokine genes with the development of different complications, no single nucleotide polymorphism (SNP) genotyping in non-HLA genes is currently used for decision making in routine clinical practice. Many of these polymorphisms have been associated with GVHD or other complications (5, 6), however, the identification of a unique polymorphism of a single gene does not have the sensitivity and specificity required for a reliable prediction of these post-transplant complications. The ideal approach would then be the combined use of several of them, along with clinical variables, to construct a predictive model. Predictive models are designed to anticipate a response variable and have become useful tools in improving the diagnostic and prognostic use of biomarkers. In this context, in recent years several groups have developed different predictive models, including clinical and genetic variables. Kim et al. built a risk model incorporating polymorphisms and clinical markers, which allowed for improved risk stratification for acute GVHD (aGVHD), TRM, overall survival (OS) and relapse-free survival (7, 8). Paczesny et al. developed a protein panel of four cytokines measured in plasma for the diagnosis of aGVHD, and another panel for patient stratification based on the risk of chronic GVHD (cGVHD) (9). Our group developed several clinical-genetic predictive models for GVHD applying a complex estimation method, the least absolute shrinkage and selection operator (LASSO) procedure (10).

In order to further gain knowledge on new genetic variants not previously described and improve our previously published predictive models, we designed a 132-gene next generation sequencing (NGS) panel (including coding and non-coding regions) to identify new polymorphisms in genes related to the immune response, specifically cytokines, which may be associated with the development of post-transplant complications.

## 2 Materials and methods

### 2.1 Study design

We consecutively selected all patients who had received an HLA-identical sibling-donor allo-HSCT in our center between 2000 and 2015 and for whom we had DNA samples available for NGS analysis. GVHD prophylaxis included cyclosporine A and methotrexate for all patients.

Those patients who died or relapsed without having developed GVHD before day +100 for aGVHD (n=2) or day +180 for cGVHD (n=28) were censored. Those patients who died due to causes not related to transplant toxicity were also censored (n=20), and in our analysis of relapse, patients who died during the first year after transplantation, without having relapsed, were also excluded (n=13).

Clinical variables considered were donor and recipient sex, donor and recipient age, underlying disease, stem-cell source, conditioning regimen, prior radiation therapy and previous HSCT (**Table 1**).

**Table 1:** Patient and donor characteristics.

Characteristic	Whole cohort (n=90)
**Recipient age** (years). Median (range)	44 (13–65)
**Donor age** (years). Median (range)	44 (11–73)
**Recipient sex** (male/female)	60/30
**Donor sex** (male/female)	48/42
Diagnosis, n (%)	
Acute myeloid leukemia Non-Hodgkin lymphoma Acute lymphoblastic leukemia Myelodysplastic syndrome Multiple myeloma Hodgkin’s lymphoma Others*	29 (32.2) 24 (26.7) 18 (20) 8 (8.9) 4 (4.4) 3 (3.3) 4 (4.4)
Stem cell source, n (%)	
PB BM	85 (94.4) 5 (5.6)
Conditioning regimen, n (%)	
Myeloablative Reduced-intensity conditioning	53 (58.9) 37 (41.1)
**Previous radiation therapy (TBI), n (%)**	16 (17.8)
**Previous autologous transplant, n (%)**	1 (1.1)

PB, Peripheral blood; BM, Bone marrow; TBI, Total body irradiation. *Others: aplastic anemia, chronic lymphocytic leukemia, chronic myeloid leukemia.

The local ethics committee approved the study, and all recipients and donors provided written informed consent in accordance with the Declaration of Helsinki.

### 2.2 Posttransplant evaluation

Post-transplant complications analyzed were grade II-IV aGVHD, III-IV aGVHD, cGVHD, moderate-severe cGVHD, TRM, relapse and OS.

GVHD classification and clinical data collection were performed at the moment of GVHD diagnosis by the attending physician following the 1994 Consensus Conference on aGVHD grading and the National Institutes of Health criteria for diagnosis and staging of cGVHD.

In the case of TRM, we have considered TRM every death that occurs while the patient is in remission, any death not attributable to relapse.

### 2.3 Genotyping

Pre-transplant samples were selected, mostly peripheral blood. The selected samples were in complete remission as the objective was to identify germline variants (polymorphism). Subsequently, genomic DNA was purified automatically (Maxwell^®^ 16 Blood DNA Purification Kit; Promega, Madison, Wisconsin) following manufacturer instructions. The DNA extracted was frozen at -80°C in our biobank (ISCIII N°C.0000915).

We designed a custom panel of 132 cytokine genes that included 73 interleukin and 59 chemokine genes (**Supplementary Table 1**). The probes were designed to detect coding and non-coding regions, namely untranslated regions (UTR), splicing (± 1,2 base pairs), and upstream and downstream regions (± 200 base pairs).

Libraries were prepared using a capture gene panel according to the manufacturer’s protocol (Agilent, Santa Clara, California). Paired-end sequencing (2×101 bp) was performed using the Illumina HiSeq platform (Illumina, San Diego, California). FASTQ files were aligned against the human reference genome (GRCh38/hg38 version) using the Burrows Wheeler Alignment tool v0.7.15-r1140. Variant calling and indel-realignment were performed using a combination of two different algorithms: GATK and VarScan. GeneSystems software (Sistemas Genómicos, Valencia, Spain) was used for variant annotation in order to provide the infrastructure and interface for bioinformatic analysis. Identified variants were annotated using the Ensembl database, population databases (the Exome Aggregation Consortium and 1000 Genomes), and specific variant databases (ClinVar, Catalogue of Somatic Mutations in Cancer (COSMIC). The population bases GenomAD and 1000 Genomes were used to determine population frequency (Minor Allele Frequency, MAF).

All polymorphisms located in coding sequences, splicing (± 1,2 base pairs), UTR and upstream and downstream regions (± 200 base pairs) were analyzed. Non-synonymous variants with ≥30X depth in the canonical isoform, a variant allele frequency (VAF) greater than 40%, a MAF ≥1% and representing at least 5% of our cohort were selected. From the total number of polymorphisms (genetic variants) analyzed, we selected those in which the presence of the minor variant (allele) is associated with the development of a complication.

### 2.4 Statistical analysis

Given the high dimensionality of the data and the limited sample size, conventional methods were not flexible enough to find discriminative features. To address this, we proposed Bayesian probabilistic methods to manage uncertainty while performing dimensionality reduction.

Data analysis consisted of two main steps: feature selection and classification or prediction. To address the first step, we developed a novel probabilistic variant of the Logistic Regressor, incorporating L1 regularization, referred to as the Bayesian Logistic Regression (BLR) model. The proposed BLR model follows a Variational Autoencoder (VAE) approach, in which the weight vector is approximated through a multivariate Gaussian distribution. Additional details of the BLR model and VAE approach can be found in the **Supplementary Material**.

In the classification step, we wanted to account for the potential non-linear relationships between the selected input variables and the complications. As the BLR is limited in its ability to capture non-linear relationships, it was only utilized for the feature selection stage. As an alternative, a probabilistic non-parametric kernel model, specifically a Gaussian Process (GP) classifier, was used to generate predictions. Additional details can be found in the **Supplementary Material**.

### 2.5 Predictive models

Taking into account the number of variables, BLR models were used to select the most important variables involved in each complication. Initially, the 15 most relevant genetic/clinical variables were selected. The strategy implemented was to select those predictive models that obtained the highest area under the curve (AUC), i.e. the greatest predictive power, with the lowest number of variables (**Supplementary Table 3**). Depending on the presence of the variables selected for each model, a score was calculated for each patient. Subsequently, taking into account sensitivity, specificity, false positives, and false negatives (in both cases setting the limit at 15%), the best cutoff point was selected for the subsequent stratification of patients into high or low risk according to the model selected for each post-transplant complication (**Supplementary Table 4**).

The selected variants were represented in **Tables 2**–**8**, in these tables display the following characteristics:

**Table 2:** Genetic risk score for aGVHD II-IV.

Variable	Polymorphism	Genomic Localization	D/R	Mean weight	Std Mean weight	Var Mean	Std Var Mean
**Age**	–	-	D	0.71	0.05	0.24	0.13
***CCL25***	rs11671930	*Upstream*	R	0.46	0.03	0.19	0.08
**Age**	–	–	R	0.36	0.03	0.24	0.07
***IL26***	rs2068016	*Upstream*	R	0.35	0.03	0.17	0.06
***CXCL13***	rs1052563	3’UTR	R	0.34	0.03	0.16	0.05
***IL2RA***	rs12722485	*Upstream*	D	0.33	0.02	0.16	0.06
***CXCR4***	rs2680880	5’UTR	D	0.31	0.03	0.12	0.05

D, Donor; R, Recipient; STD, Standard deviation; Var, Variance.

**Table 3:** Genetic risk score for aGVHD III-IV.

Variable	Polymorphism	Genomic Localization	D/R	Mean weight	Std Mean weight	Var Mean	Std Var Mean
***IL12RB1***	rs3746190	3’UTR	R	0.43	0.04	0.17	0.08
***IL17A***	rs3819024	*Upstream*	R	0.35	0.03	0.13	0.06
***IL17RA***	rs4819962	3’UTR	R	0.32	0.03	0.14	0.05
***IL21R***	rs961914	5’UTR	D	0.31	0.02	0.15	0.05
***CCL25***	rs1129763	*Missense*	R	0.28	0.02	0.14	0.04
***CXCR2***	rs1126580	3’UTR	R	0.27	0.02	0.13	0.07
***CXCL16***	rs76152703	3’UTR	D	0.26	0.02	0.14	0.04
***IL17D***	rs9579928	*Upstream*	D	0.26	0.02	0.12	0.04
***IL2RA***	rs12722602	3’UTR	D	0.26	0.02	0.13	0.04

D, Donor; R, Recipient; STD, Standard deviation; Var, Variance.

**Table 4:** Genetic risk score for cGVHD.

Variable	Polymorphism	Genomic Localization	D/R	Mean weight	Std Mean weight	Var Mean	Std Var Mean
***IL2RA***	rs12722485	**Upstream**	R	0.44	0.04	0.21	0.08
***IL7R***	rs10063294	**3’UTR**	R	0.41	0.03	0.16	0.07
***XCR1***	rs2371	**Upstream**	D	0.30	0.03	0.12	0.05
***IL3RA***	rs17883366	**Missense**	R	0.29	0.03	0.13	0.05

D, Donor; R, Recipient; STD, Standard deviation; Var, Variance.

**Table 5:** Genetic risk score for moderate-severe cGVHD.

Variable	Polymorphism	Genomic Localization	D/R	Mean weight	Std Mean weight	Var Mean	Std Var Mean
***IL7R***	rs10063294	3’UTR	R	0.46	0.04	0.20	0.10
***IL7R***	rs72742450	3’UTR	R	0.44	0.03	0.17	0.06
***IL17RC***	rs279549	*Missense*	R	0.38	0.03	0.17	0.06
***CXCR6***	rs3774639	*Upstream*	D	0.36	0.03	0.14	0.08
***IL10RA***	rs4252243	*Upstream*	R	0.34	0.03	0.16	0.04
***IL25***	rs7145551	*Upstream*	D	0.31	0.03	0.13	0.05
***IL12RB1***	rs11575934	*Missense*	R	0.30	0.03	0.13	0.04
***IL7***	rs6997891	*Upstream*	D	0.27	0.02	0.14	0.04
***IL11***	rs2298885	3’UTR	D	0.26	0.03	0.12	0.04

D, Donor; R, Recipient; STD, Standard deviation; Var, Variance.

**Table 6:** Genetic risk score for TRM.

Variable	Polymorphism	Genomic Localization	D/R	Mean weight	Std Mean weight	Var Mean	Std Var Mean
***IL20RB***	rs835634	*Upstream*	R	0.36	0.03	0.14	0.06
***IL20RB***	rs835632	*Upstream*	R	0.35	0.03	0.14	0.06
***IL12RB1***	rs404733	3’UTR	D	0.31	0.02	0.13	0.06
***CXCL11***	rs9994667	3’UTR	R	0.30	0.03	0.14	0.05
***IL15RA***	rs2387089	*Upstream*	D	0.29	0.02	0.15	0.06
***CXCL2***	rs9131	3’UTR	R	0.28	0.02	0.14	0.05

D, Donor; R, Recipient; STD, Standard deviation; Var, Variance.

**Table 7:** Genetic risk score for relapse.

Variable	Polymorphism	Genomic Localization	D/R	Mean weight	Std Mean weight	Var Mean	Std Var Mean
***IL21R***	rs8060368	*Upstream*	R	0.50	0.04	0.20	0.09
***CCL15***	rs2293788	5’UTR	D	0.45	0.03	0.20	0.07
***CCL21***	rs11574915	5’UTR	R	0.37	0.03	0.17	0.05
***IL21R***	rs961914	5’UTR	R	0.35	0.02	0.17	0.05
***CXCL11***	rs9994667	3’UTR	R	0.34	0.03	0.14	0.06
***IL21R***	rs2189521	5’UTR	R	0.34	0.02	0.12	0.06
***CXCR4***	rs2680880	5’UTR	D	0.30	0.03	0.13	0.05
***CCL15***	rs2293788	5’UTR	R	0.28	0.01	0.15	0.04
***CCL15***	rs41508645	5’UTR	R	0.28	0.02	0.15	0.03
***CCR10***	rs3760384	*Upstream*	R	0.28	0.02	0.11	0.07

D, Donor; R, Recipient; STD, Standard deviation; Var, Variance.

**Table 8:** Genetic risk score for OS.

Variable	Polymorphism	Genomic Localization	D/R	Mean weight	Std Mean weight	Var Mean	Std Var Mean
***CXCL11***	rs9994667	3'UTR	R	0.55	0.05	0.19	0.10
***CCL21***	rs11574915	5'UTR	R	0.45	0.04	0.20	0.06
***CCL16***	rs2063979	3'UTR	D	0.34	0.02	0.16	0.05
***CCL16***	rs146038760	3'UTR	D	0.34	0.02	0.17	0.05
***IL-10RB***	rs1058867	3'UTR	R	0.33	0.025	0.13	0.056
***CCL25***	rs2032887	*Missense*	D	0.32	0.03	0.15	0.06
***IL-12RB1***	rs3746190	3'UTR	D	0.32	0.02	0.14	0.06

D, Donor; R, Recipient; Std, Standard deviation; Var, Variance.

Mean weight: Mean over partitions of the inferred mean in absolute value.
Std Mean weight: Standard deviation over partitions of the inferred mean.
Var Mean: Mean over partitions of the inferred variance.
Std Var Mean: Standard deviation over partitions of the inferred variance.

### 2.6 Functional analysis of the genes included in the selected predictive models

An enrichment analysis of the genes included in the models ultimately selected was conducted using the Enrichr web-based software application in order to establish common functions and establish a functional relationship between them.

## 3 Results

### 3.1 Clinical data

Of the global cohort, 51/88 (58%) of patients presented grade II-IV and 27/88 (30.6%) III-IV aGVHD. Additionally, 39/63 (62%) and 25/61 (41%) developed cGVHD and moderate-severe GVHD respectively. Regarding TRM, 22/70 (31,4%) patients presented. Finally, 17/81 (23%) and 44/90 (49%) patients relapse or died respectively.

The cumulative incidence (CI) of grade II-IV and III-IV aGVHD at 100 days post-transplant was 56.7% and 23.3%, respectively. Cumulative incidence of cGVHD and moderate-severe cGVHD (mod-sev cGVHD) at three years post-transplant was 43.3% and 27.8%, respectively. Three-year CI for TRM was 24.4%, and 2-year OS was 60%.

The median times and the survival plot of each complication are shown in **Supplementary Figure 1**:

### 3.2 Variant data

**Figure 1:**
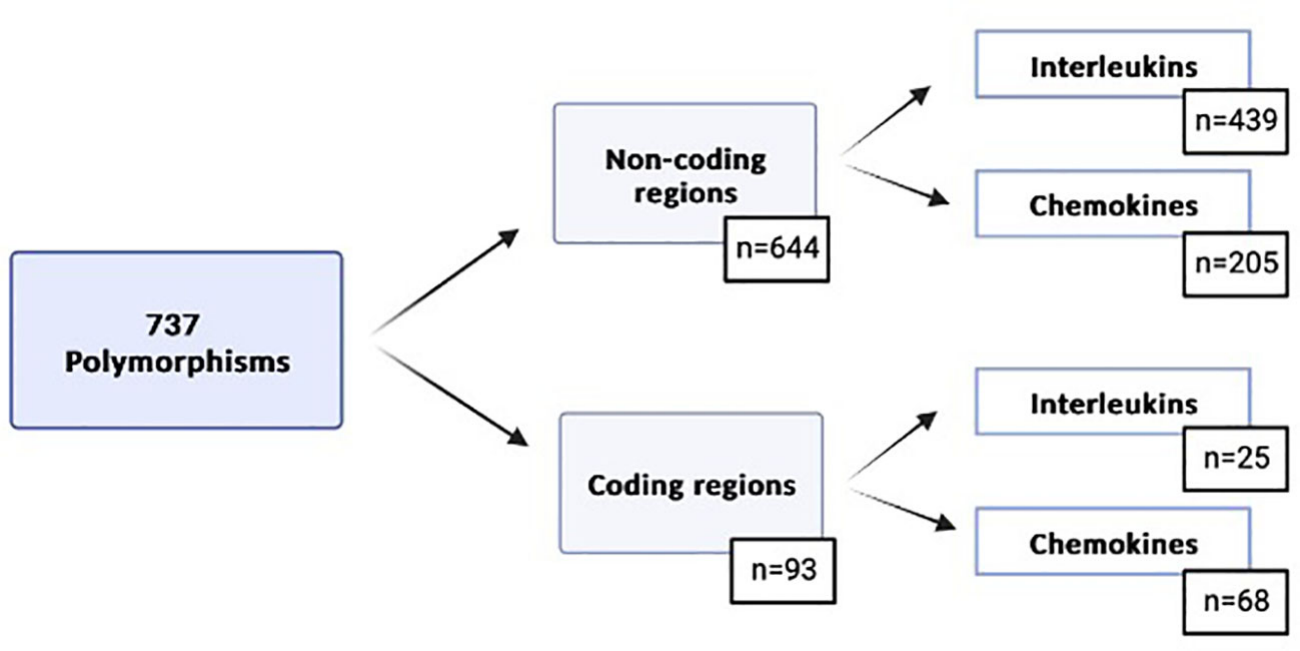
Genetic variables selected based on the previously defined filters.

Using the filters previously defined in Material and Methods, 737 polymorphisms in the 119 genes were detected in 90 donor-recipient pairs (**Supplementary Table 2**, **Figure 1**). Of these polymorphisms, 93 (12.6%) were located in coding regions, and 644 (87.4%) in non-coding regions: 3’ and 5’ UTR, splicing, upstream and downstream regions.

### 3.3 Predictive models for post-transplant complications

Different predictive models, including clinical variables and 737 genetic variants, were built to anticipate the development of post-transplant complications such as GVHD, TRM, relapse and OS following allo-HSCT.

Initially, the 15 most relevant genetic/clinical variables were selected to reduce the dimensionality of the data (**Supplementary Table 3**). Of these initial 15 variables, the final variables for predictive models were selected taking into account the AUC and the number of variables. The models ultimately obtained for each of the post-transplant complications (aGVHD II-IV, aGVHD III-IV, cGVHD, mod-sev cGVHD, TRM, relapse and OS) are shown in **Tables 2**–**8**. Finally, 41 polymorphisms in 30 cytokine genes were selected (17 interleukins and 13 chemokines). Of these polymorphisms, 5 (12.2%) were located in coding regions, and 36 (87.8%) in non-coding regions.

**Figure 2:**
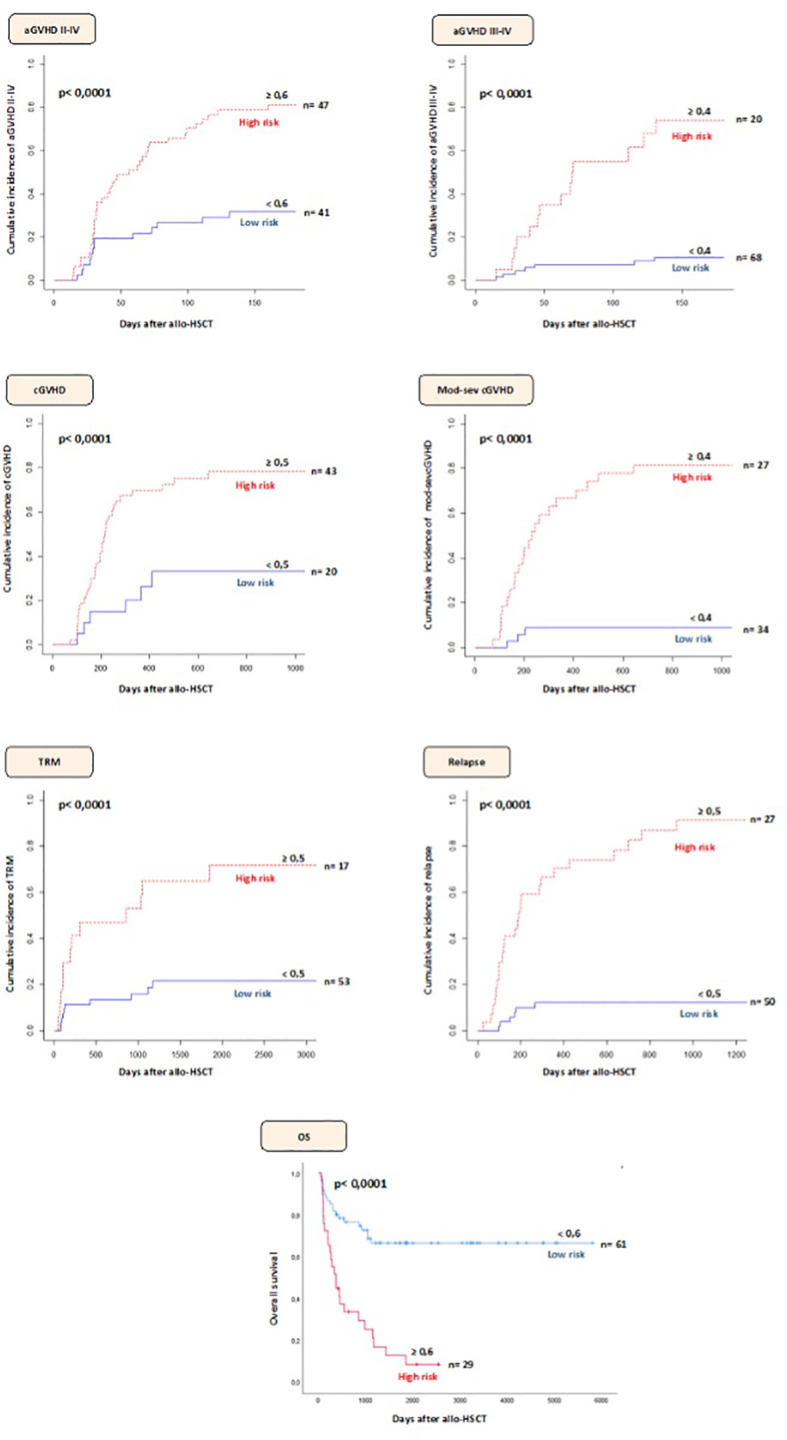
Stratification of patients at high/low-risk of developing complications after allo-HSCT according to the selected predictive model and cut-off point.

Based on the sensitivity and specificity data, as well as the ratio of false positives and false negatives (a limit of 15% was established), cut-off points were selected to classify the patients at high or low-risk for the development of each post-transplant complication (**Supplementary Table 4**). Finally, the cumulative incidence of each complication according to patient risk stratification (**Figure 2**).

#### 3.3.1 GVHD

##### 3.3.1.1 aGVHD

###### 3.3.1.1.1 Grade II-IV aGVHD

The predictive model selected consisted of seven variables in 5 genes (*CCL25, IL-26, CXCL13, IL-2RA, CXCR4*) (**Table 2**) and achieved AUC: 0.81, sensitivity: 74.77% and specificity: 73.53%. The only clinical variables selected were donor and recipient age. We calculated the II-IV aGVHD risk score for each patient to test the usefulness of the model in identifying patients at a high-riskof experiencing an II-IV aGVHD following allo-HSCT. At 180 days after allo-HSCT, 80.8% of patients with a high-risk score experienced II-IV aGVHD compared to 31.7% of those with a low-risk score (**Figure 2**).

###### 3.3.1.1.2 Grade III-IV aGVHD

A model (with AUC: 0.81; sensitivity: 65.29% and specificity: 91.09%) consisting of nine genetic variables (*IL-12RB1, IL-17A, IL-17RA, IL-12R, CCL25, CXCR2, CXCL16, IL-17D, IL-2RA*) was selected (**Table 3**). According to this model, we observed that 70% of the patients classified as high-risk developed this complication while only 10.3% of patients classified as low-risk showed III-IV aGVHD at 180 days post-transplant (**Figure 2**).

##### 3.3.1.2 cGVHD

###### 3.3.1.2.1 cGVHD

The model developed (AUC: 0.73; sensitivity: 83.28% and specificity: 58.99%) consisted of four genetic variables (*IL-2RA, IL-7R, XCR1, IL-3RA*) (**Table 4**). Based on the selected model, 76.7% of the patients with a high-risk score developed cGVHD at two years post-transplant. However, 30% of patients classified as low-risk also developed cGVHD (**Figure 2**).

###### 3.3.1.2.2 Moderate-severe cGVHD

Based on the number of variables and their associated weight, a genetic model (AUC: 0.88; sensitivity: 87.88% and specificity: 84.34%) composed of nine polymorphisms in 8 genes (*IL-7R, IL-17RC, CXCR6, IL-10RA, IL-25, IL-12RB1, IL-7, IL-11*) was selected (**Table 5**). We calculated the moderate-severe cGVHD risk score for each patient. Of the participants, 81.4% with a high-risk score experienced moderate-severe cGVHD compared to 14.8% of those with a low-risk score at two years after allo-HSCT (**Figure 2**).

#### 3.3.2 TRM

The model selected for TRM (AUC: 0.74; sensitivity: 54.79% and specificity: 88.93%) was composed of six polymorphisms in 5 genes (*IL-20RB, IL-12RB1, CXCL11, IL-15RA, CXCL2*) (**Table 6**). Based on the stratification of patients into high or low-risk based on the cutoff point it was observed that the CI of TRM in patients classified as high-risk was 47.1% at two years post-transplant. In contrast, the CI of low-risk patients was 13.2% (**Figure 2**).

#### 3.3.3 Relapse

This predictive model (AUC: 0.93; sensitivity: 79.06% and specificity: 90.61%) included ten polymorphisms in 6 genes (*IL-21R, CCL15, CCL21, CXCL11, CXCR4, CCR10*) (**Table 7**). Based on the selected model, 81.5% of high-risk patients had relapsed at two years post-transplant. In contrast, 12% of low-risk patients relapsed at two years after allo-HSCT (**Figure 2**).

#### 3.3.4 Overall survival

Finally, seven genetic variables in 6 genes (*CXCL11, CCL21, CCL16, IL-10RB, CCL25, IL-12RB1*) were selected for this model (AUC: 0.78; sensitivity: 57.39% and specificity: 86.81%; **Table 8**). When the model selected was applied, it was observed that at five years post-transplant 17.3% of the patients classified as high-risk were still alive compared to 68.6% of the low-risk patients (**Figure 2**).

### 3.4 Enrichment analysis

Considering the genes included in the predictive models for post-transplant complications, 41 polymorphisms in 30 cytokines. Four signaling pathways functionally associated with the genes were obtained by enrichment analysis using web-based Enrichr software (**Table 9**) with a p-value of 2.5*10^-56^, 1.48*10^-27^, 4.04*10^-18^ and 4.97*10^-9^, respectively.

**Table 9:** Functional relationship between the genes selected for each post-transplant complication and the signaling pathway in which they participate as well as predictive models of the complications in which each gene has been selected.

	Signaling pathway				Predictive models for complications						
	Cytokines	Chemokines	JAK/STAT	IL17/T_H_17	II-IV aGVHD	III-IV aGVHD	cGVHD	Mod-sev cGVHD	TRM	Relapse	OS
***IL-7***											
***IL-11***											
***IL-17A***											
***IL-17D***											
***IL-25***											
***IL-26***											
***IL-2RA***											
***IL-3RA***											
***IL-7R***											
***IL-10RA***											
***IL-10RB***											
***IL-12RB1***											
***IL-15RA***											
***IL-17RA***											
***IL-17RC***											
***IL-20RB***											
***IL-21R***											
***CCL15***											
***CCL16***											
***CCL21***											
***CCL25***											
***CXCL2***											
***CXCL11***											
***CXCL13***											
***CXCL16***											
***CCR10***											
***CXCR2***											
***CXCR4***											
***CXCR6***											
***XCR1***											

All genes selected were included in the cytokine signaling pathway. Another signaling pathway selected by Enrichr was JAK/STAT, in which 13/30 cytokines, specifically interleukins (43.3%), were included. Finally, the IL-17/TH17 signaling pathway consisted of 6/30 cytokines (20%), including five interleukins and one chemokine.

## 4 Discussion

Allo-HSCT may be a successful curative treatment for hematological malignancies mainly due to GVL. Despite current knowledge on the pathophysiology of allo-HSCT, it continues to be a complex procedure in which a large number of patients experience related complications, including disease relapse, which represents the leading cause of treatment failure, and GVHD. Approximately 40% of patients develop GVHD; consequently, GVHD and associated infectious complications contribute to transplant-related morbidity and mortality (11).

There are several known risk factors that have an impact on the results of allo-HSCT, including HLA histocompatibility, the hematopoietic stem cell source, sex/age disparity between donor and recipient, conditioning regimen, underlying disease, etc. (12). However, the use of these clinical variables is not enough to accurately identify those patients who are at higher risk of developing post-transplant complications.

Recently, it has been shown that genetic variability (polymorphisms) in non-HLA genes also affects the outcome of allo-HSCT (13). In this context, there is evidence that polymorphisms in cytokine genes, which usually alter the expression or function of these proteins, impact the immune response that occurs in GVHD and could therefore be used as biomarkers to anticipate the risk of developing these complications (8, 9, 14–16). Therefore, it is important to build risk models in which several polymorphisms and clinical variables are included in order to anticipate these complications more accurately. In order to extend our knowledge to new genetic variants not previously described, we designed an NGS panel of 132 genes (including coding and non-coding sequences) to identify new polymorphisms in genes related to the immune response, specifically cytokines, which could be related to the development of post-transplant complications.

A total of 737 polymorphisms were selected from the custom panel genes. Of these, 41 polymorphisms were included in the predictive models, of which 87.8% were located in non-coding regions (UTR, upstream and downstream). It is interesting to note that most of the selected genetic variables had not been previously reported. **Supplementary Table 5** provides a brief summary of the biological function of each of the selected genes, as described in the literature. Although it is not yet possible to specify the exact effect of the polymorphisms on the function of each gene, it is important to provide context for the function of each of these genes in the immune system.

Therefore, the non-coding regions could be of significant importance as these are usually related to increased or decreased protein expression. Although functional studies are not yet available, the selected variables could be related to increased expression of the proinflammatory protein, as occurs with many of the polymorphisms described, or decreased expression of an anti-inflammatory protein for which they encode and therefore influence the risk of developing different post-transplant complications.

### 4.1 GVHD models

**Figure 3:**
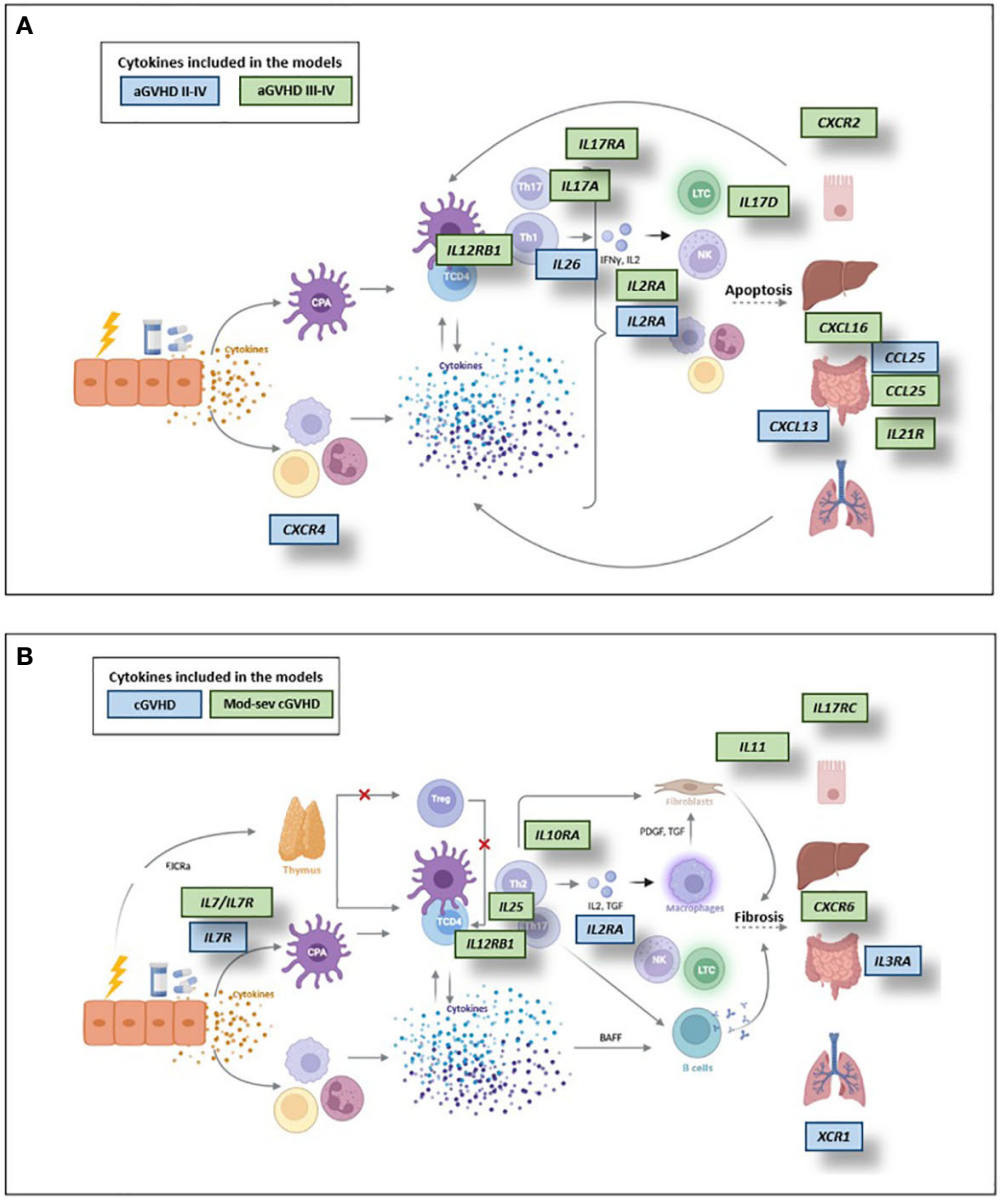
Cytokines included in the predictive models of aGVHD **(A)** and cGVHD **(B)**. APC, Antigen-presenting cells; TCD4, CD4 T cells; BAFF, B-cell activating factor of the tumor necrosis factor family. Created with Biorender.com.

**GVHD models** Interleukins related to the activation, proliferation and differentiation of immune system cells involved in the development of GVHD were included in acute and chronic GVHD models. In addition, different chemokines responsible for driving these cells to the sites of inflammation were included (**Figure 3A**). IL-12RB1 is a CD4^+^ T cell receptor that, depending on the binding of the IL-12 or IL-23 ligand, leads to a differentiation to a Th1 or Th17 phenotype (17), characteristic of aGVHD and cGVHD, respectively, that was selected for the aGVHD and cGVHD models. The IL-2RA receptor, which is responsible for the proliferation of T cells involved in GVHD and which in soluble form has been reported to be increased in stages of immune activation (18), was also included in these models. Specifically, the aGVHD model included interleukins such as IL-17A, IL-17RA and IL-26 related to the synthesis of proinflammatory cytokines (19, 20), which may be involved in the development of this complication. CCL25, although described in inflammatory diseases, is also associated with the proliferation of regulatory T cells (Tregs) that inhibit GVHD (21, 22). IL-21R has been reported as responsible for the proliferation and differentiation of lymphoid cells, but there are already studies that demonstrate that blocking IL-21/IL-21R in the gut increases Tregs, thereby inhibiting GVHD (23). Regarding cGVHD, it is worth noting that the chemokines selected in the predictive models have already been described in the literature as related to the development of GVHD. The interleukins IL-7/IL-7R, related to survival, proliferation and activity of T and B cells (24); as well as IL-25, which is related to TH2 activation but inhibits TH17 (25), T cells related to cGVHD, were also included (**Figure 3B**).

Notably, a greater number of genes were selected in the more severe GVHD models. This may be explained by the fact these could be expected to involve a more inflammatory context affecting various organs and thus producing a greater severity of GVHD. This is consistent with the selected models for severe GVHD obtained in a previous study (8).

Regarding the genetic model for TRM, all interleukins selected signaled through the JAK/STAT pathway, leading to the synthesis of proinflammatory cytokines. These cytokines could lead to an inflammatory environment that can affect TRM due to tissue damage. This is further enhanced by the chemokines selected for this model, which are related to the migration of immune system cells to inflammatory sites.

For the relapse model, three polymorphisms were selected in a single interleukin, IL-21R, which through its signaling leads to the proliferation and differentiation of lymphoid cells (26, 27). Thus, it could positively or negatively regulate immune responses. It worth noting that mainly chemokines were selected in this model (7/10). This could be explained as proposed in a study of pediatric patients diagnosed with acute lymphoblastic leukemia where the expression of chemokines and their receptors is increased in relapsed patients. In this study the authors propose that the cells secrete chemokines that recruit those leukocytes that express the specific receptor. Thus, tumor cells that secrete and/or respond to chemokines would have a selective advantage and may show resistance to chemokine therapy (28).

Finally, regarding the OS model, IL-10RB signaling exerts anti-inflammatory functions (29); hence we must identify the effect of the polymorphism selected on the expression of this gene. In turn, signaling of the other interleukin included, IL-12RB1, leads to the synthesis of IFN-γ, which may have an antitumor role in the activation of effector T cells, which will destroy tumor cells. However, it may also have a protumor role, involving proliferative and antiapoptotic signals (30). The chemokines selected are related to the migration and differentiation processes of different cells in the immune system. It is likely that these chemokines are able to recruit different types of immune cells in the tumor which, in turn, can modulate tumor growth and metastasis, as has been demonstrated in the literature (31).

An analysis of enrichment with the Enrichr application (32) found that in addition to the cytokine and chemokine signaling pathway, as expected, two signaling pathways represented in the inflammatory context were also selected: the IL17/TH17 and JAK/STAT signaling pathways. TH17 cells are CD4^+^ T cells that produce proinflammatory cytokines such as IL-17A, IL-17F, IL-21, IL-22, TNF-α, G-CSF and some chemokines. IL-17 is related to innate immunity and inflammation and relates T cell activation to neutrophil mobilization and activation (33). Several studies provide evidence of the role of IL-17 in GVHD, demonstrating that activated TH1 and TH17 lymphocytes secrete proinflammatory cytokines that lead to apoptosis of cells in target tissues in GVHD, primarily in the intestine, liver and skin (34, 35). The JAK/STAT signaling pathway regulates the activation of immune cells related to GVHD, including APCs, T cells, neutrophils and B cells (36, 37). It is therefore involved in regulating cell activation, proliferation, migration and cytokine production, increasing the severity of GVHD. In this context, drugs such as ruxolitinib that inhibit this signaling pathway are already available and used for the treatment of GVHD (38).

Based on the results obtained, it should be taken into account that most of the genetic variables selected for the predictive models of each of the post-transplant complications were located in non-coding regions, and that most of these polymorphisms identified for their clinical relevance (association with post-transplant complications) are not described in the literature. Therefore, it will be important in the future to conduct functional studies to determine whether these genetic variables produce changes in protein expression. In addition, these models should be validated with a larger sample size and in other HSCT regimens.

In conclusion, the incorporation of these predictive models in the management of transplanted patients may contribute to optimize the treatment and improve the outcomes of these patients in the context of a personalized precision medicine.

## Data Availability

The datasets presented in this study can be found in online repositories. The names of the repository/repositories and accession number(s) can be found in the article/**Supplementary Material**. The datasets analyzed for this study can be found in the repositorio institucional de la consejería de sanidad de la comunidad de madrid: https://repositoriosaludmadrid.es//handle/20.500.12530/87923.

## Supplementary material

Supplementary Table 1 Genes included in the designed NGS panel, corresponding to interleukins and chemokines.

Supplementary Table 2 Genetic variables obtained after application of the filters described in the Patients and Methods section for the analysis of the designed NGS panel. Cr: Chromosome; Ref: Most frequent allele; Var: Minority variant allele; EUR MAF: Minority allele frequency in Europeans.

Supplementary Table 3 AUC of the 15 most relevant variables included in the selected predictive models for post-transplant complications. D: Donor; R: Recipient; STD: standard deviation; GVHD: graft-versus-host disease; aGVHD: acute graft-*versus*-host disease; cGVHD: chronic graft-versus-host disease; mod-sev cGVHD: moderate-severe cGVHD; TRM: transplant-related mortality; OS: overall survival.

Supplementary Table 4 Relationship of the characteristics of the different cut-off points for the stratification of patients for each of the post-transplant complications. aGVHD: acute graft-versus-host disease; cGVHD: chronic graft-*versus*-host disease; mod-sev cGVHD: moderate-severe cGVHD; TRM: transplant-related mortality; OS: overall survival; STD: standard deviation; S: sensitivity; E: specificity. The cut-off points selected for stratification of patients at high/low-risk of developing each complication according to the selected model are identified in bold.

Supplementary Table 5 Function and polymorphisms associated with each post-transplant complication of the genes selected in the present study. GVHD: Graft-versus-host disease; aGVHD: acute graft-versus-host disease; cGVHD: chronic graft-versus-host disease; mod-sev cGVHD: moderate-severe cGVHD; TRM: Transplant-related mortality; OS: Overall survival; IL: Interleukin; TH: T-helper T lymphocyte. Proinflammatory cytokines are identified in yellow, and chemokines included in the GVHD models are identified in orange.
