# Supplementary material for: Identification of predictive models including polymorphisms in cytokines genes and clinical variables associated with post-transplant complications after identical HLA-allogeneic stem cell transplantation
Source: Front Immunol. 2024 Aug 23;15:1396284. doi: 10.3389/fimmu.2024.1396284 (PMC11377344; doi:10.3389/fimmu.2024.1396284)
Supplement: Supplementary file 6 [file DataSheet1.docx]

# Introduction

This supplementary material provides a detailed description of the model we have developed to analyze the data presented in the main document: the Bayesian Logistic Regression (BLR) model. It is a deep probabilistic version of a Logistic Regression (LR) model that follows a Variational Autoencoder (VAE) [1] approach, which is flexible enough to deal with datasets that present a great unbalance between the number of observations and number of variables.

In the following sections, we briefly introduce the VAE, which is the base of the proposed method. Then, we define the BLR and explain the inference process, as well as its architecture.

# Model description

## VAE description

Autoencoders are Neural Networks (NNs) that infer a low-dimensional representation of the input data in a nonlinear unsupervised way, minimizing the reconstruction error. In VAEs, this compact representation is learnt by means of Variational Inference (VI), assuming the observed data is generated from an underlying (latent) low-dimensional probability distribution. The goal is to describe this latent (encoded) representation ***z*** given the observed (decoded) data ***x***, i.e., the goal is to infer the posterior probability distribution *p*(***z***|***x***), which can be obtained applying Baye’s Theorem (1).

*p*(**z**|**x**) =

*p*(**x**|**z**)*p*(**z**) *p*(**x**)

(1)

In the vanilla VAE, the prior distribution of the latent variable *p*(***z***) (2) and the likelihood

*p*(***x***|***z***) (3) are assumed Gaussian

*p*(***z***) = N(**0***,* I)*,* (2)

*p*(***x***|***z***) = N(***µθ*** (***z***)**Σ*_θ_***(***z***))*,* (3)

where the mean vector ***µθ*** (***z***) and diagonal covariance matrix **Σ*_θ_***(***z***) are obtained by means of a NN (decoder) with parameters ***θ***. Note this model is i.i.d.

As the marginal distribution *p*(**x**) in (1), given by

*p*(***x***) = ∫ *p*(***z***)*p*(***x***|***z***)*d****z****,* (4)

is intractable, it is not possible to obtain *p*(**z**|**x**) by exact inference. Instead, the posterior distri- bution is approximated by means of VI assuming a Gaussian variational family

*q*(***z***|***x***) = N(***µϕ***(***x***)*,* **Σ*_ϕ_***(***x***))*,* (5)

where the mean vector ***µϕ***(***z***) and the diagonal covariance matrix **Σ*_ϕ_***(***z***) are the outputs of a NN with parameters ***ϕ***.

In order to find the distribution that better approximates the posterior *p*(***z***|***x***) among the described variational family, the Evidence Lower Bound (ELBO) (6) is maximized.

log *p*(***x***) ≥ *ELBO* = E*_q_*_(_***_z_***_|_***_x_***_)_ *p*(***x***|***z***) − D*_KL_* *q*(***z***|***x***)||*p*(***z***) (6)

As NNs are trained by means of stochastic gradient descent optimization, the negative ELBO is used as loss function. This way, the likelihood of the observed data (reconstruction term) is maximized while the posterior *p*(***z***|***x***) remains close to the defined prior *p*(***z***), as the rightmost term (the Kullback–Leibler (KL) divergence) acts a regularization term.

## BLR description

In the BLR model, we learn a vector of weights for classification instead of a compact representation

of the observed data, following the idea of a LR model. Assume a labelled {***x****_i_, y_i_*}*^N^*

*i*=1

dataset

where ***x*** is the D-dimensional feature vector and *y* is the target. We denote these variables as ***X*** = [***x***_1_*,* ***x***_2_*, ...,* ***x****_N_* ]*^T^* and ***y*** = [*y*_1_*, y*_2_*, ..., y_N_* ]*^T^* . Note that, although we assume binary labels, the model naturally handles categorical targets.

We assume a zero mean Laplacian distribution as prior for the weights **w**, which is given by

*p*(**w**) =

*D*

*d*Y=1

*Laplace*(*w_d_*|0*, b*) =

*d*Y=1

1

*exp*

2*b*

−|*w_d_*|

*b*

(7)

*D*

where *b* ∈ **R**_++_ controls the variance of the distribution.

Following the formulation of a LR model, the likelihood is given by

*N*

Y

*p*(**y**|**X***,* **w**) = *p*(*y_n_*

*n*=1

|**x***_n_*

**N**

*,* **w**) = *σ*(**w^T^x_n_**

Y

**n**=**1**

)**^y^n** **1** − *σ*(**w^T^x_n_**

) (**1**−**yn**) (8)

where *σ*(·) refers to the Sigmoid function.

Then, following Bayes’ Theorem, the posterior distribution can be expressed as

*p*(**w**|**X***,* **y**) =

*p*(**y**|**X***,* **w**)*p*(**w**)

*p*(**y**|**X**)

(9)

As the marginal distribution present in the denominator, given by

*p*(**y**|**X**) = ∫ *p*(**y**|**X***,* **w**)*p*(**w**)*d***w***,* (10)

is intractable, we approximate the posterior distribution by means of VI following a VAE approach. This way, the posterior *p*(**w**|**X***,* **y**) is approximated as a product of Gaussian distributions whose parameters (mean vectors ***µ*** and covariance matrices **Σ**) are learnt from the data using four fully connected NNs (11), following the architecture depicted in Figure 1. As the weight vector **w** is a global variable, it is obtained as a product of the local contribution of each observed data point [2].

*p*(**w**|**X***,* **y**) ≈ *q*(**w**|**X***,* **y**) = N(***µ****,* **Σ**) ∝

Y N ***µ_η_***1 (**x***_n_*)*,* **Σ*_η_***1 (**x***_n_*)

*N*

*yn* N

***µ_η_***0 (**x***_n_*)*,* **Σ*_η_***0 (**x***_n_*)

(1−*yn*) (11)

*m c m c*

*n*=1

In this expression, ***η*** refers to the parameters of the corresponding NN. Superscripts indicate the value of the label (0 or 1), while subscripts indicate whether the parameters correspond to the mean (*m*) or to the covariance (*c*) NN. Note that *q*(**w**|**X***,* **y**) critically depends on the target value in order to have a greater influence of *y* through the learning process. This way, we force the model to learn different parameters (and thus different distributions) for data with different label values. The mean and covariance of the resulting Gaussian are computed as

*N*

Σ

***µ*** = **Σ*η****yi* (***z****_i_*)^−1^

−1 Σ*N*

**Σ*η****yi* (***z****_i_*)^−1^***µη****yi* (***z****_i_*)

(12)

*c*

*i*=1

Σ*N*

**Σ** =

*i*=1

*c*

*c m*

−1

**Σ*η****yi* (***z****i*)−1

(13)

*i*=1


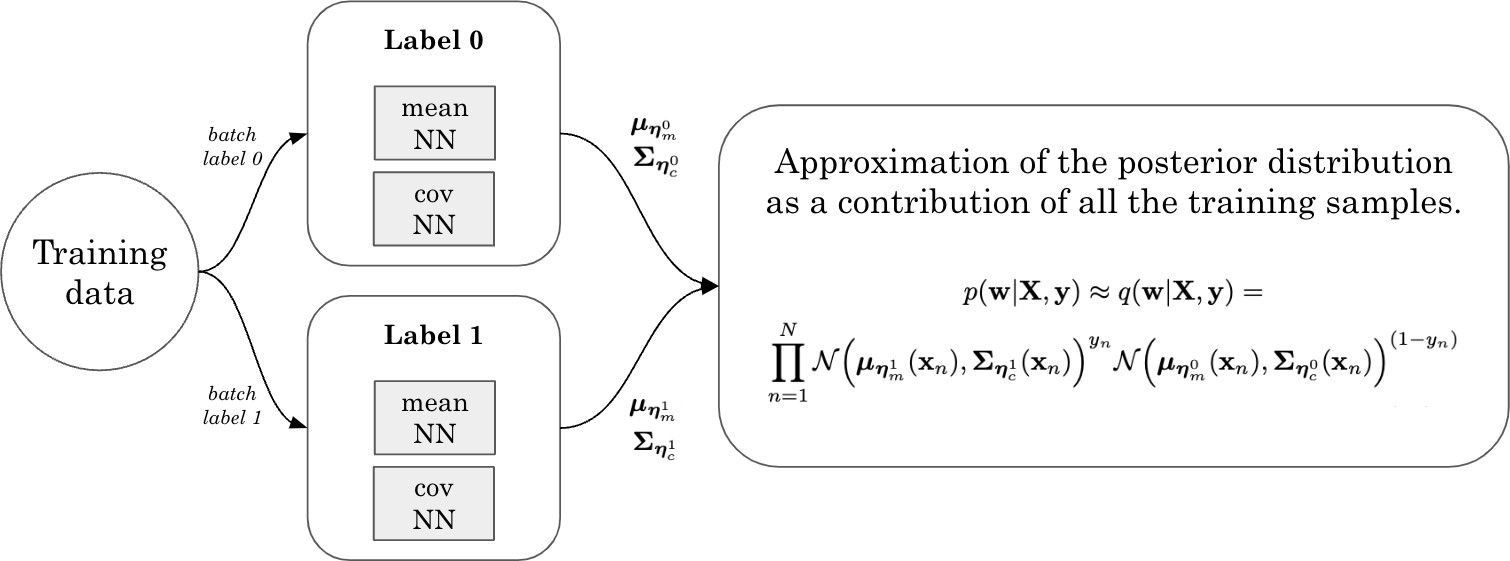


Figure 1: BLR architecture.

In order to find the best approximation of the posterior distribution among the Gaussian variational family, the ELBO is maximized. It can be expressed as

*ELBO* = ∫ *q*(**w**|**X***,* **y**) log *p*(**y**|**X***,* **w**)*p*(**w**) *d***w** = E h log *p*(**y**|**X***,* **w**)*p*(**w**) i

*q*(**w**|**X***,* **y**)

*^q^ q*(**w**|**X***,* **y**)

(14)

= E*_q_*h log *p*(**y**|**X***,* **w**)i + E*_q_*h log *p*(**w**)i − E*_q_*h log *q*(**w**|**X***,* **y**)i

and its three terms as

h i

E*_q_*

log *p*(**y**|**X***,* **w**)

h Σ*N*  i

= E*_q_*

*y_n_* log

*σ*(**w***^T^* **x***_n_*)

+ (1 − *y_n_*) log

1 − *σ*(**w***^T^* **x***_n_*)

(15)

*n*=1

E*q* h

log *p*(**w**)i

= E*_q_*

*D*

*d*=1

h Σ

1

log

2*b*

— |*w_d_*|

*b*

(16)

E*_q_*h log *q*(**w**|**X***,* **y**)i = E*_q_*h log N ***µη****m* (**X***,* **y**)*,* **Σ*_η_****c* (**X***,* **y**) i

i

2

*q*

***η****c*

***η****m*

***η****c*

(17)

= − 1 E h log det 2*π***Σ** (**X***,* **y**) + **w** − ***µ*** (**X***,* **y**) *T* **Σ** (**X***,* **y**)^−1^ **w** − ***µ***

***η****m*

(**X***,* **y**) i

Note that the prior distribution *p*(**w**) acts as a regularization term, forcing weights to take values close to zero (mean of the prior distribution). Therefore, a variable has necessarily to be informative to end with a significant associated weight. In fact, it can be seen as a probabilistic equivalent to the L1 regularization present in an standard LR model, as it is also proportional to the sum of the absolute values of the weights. In this case, the diversity term *b* controls the strictness of this regularization, as it controls the variance of the prior distribution.

Once the model is trained, it is possible to obtain predictions (class probabilities) for new observations evaluating the predictive distribution, given by

*p*(*y*^∗^|**x**^∗^) = ∫ *p*(*y*^∗^|**x**^∗^*,* **w**)*q*(**w**|**X***,* **y**)*d***w** = E*_q_*h*p*(*y*^∗^|**x**^∗^*,* **w**)i (18)

Although the model is highly flexible thanks to the VAE approach, note that this final prediction is linear, as it follows the formulation of a LR model (8).

As an alternative, a probabilistic non-parametric kernel model, specifically a Gaussian Process (GP) classifier, was used to generate predictions.

Assume a labeled dataset ${\{\boldsymbol{x}_{i}, y_{i}\}}_{i=1}^{N}$ where $\boldsymbol{x}$ is the D-dimensional feature vector and $y$ is the target. A GP is a random process where each point $\boldsymbol{x}$ is assigned a random Gaussian variable $f\left( \boldsymbol{x} \right)$ (latent function), being the joint distribution of $f\left( \boldsymbol{x} \right)$ for different values of x a zero-mean multivariate Gaussian with covariance given by a kernel $\boldsymbol{k}\left( \boldsymbol{x}_{i}, \boldsymbol{x}_{j} \right)$. In our case, we use an RBF (Radial Basis Function) kernel, which is given by

$$\boldsymbol{k}\left( \boldsymbol{x}_{i}, \boldsymbol{x}_{j} \right)=exp\left( -{\frac{\left\| \boldsymbol{x}_{i}-\boldsymbol{x}_{j} \right\|}{2\alpha^{2}}}^{2} \right)$$

being α a hyperparameter of the model.

For binary classification, these latent functions $f\left( \boldsymbol{x} \right)$ are additionally *squashed* through the logistic function $\sigma(\cdot)$ to obtain class probabilities (probability of high-risk and low-risk in our case).

$$\pi= p\left( y=1 | x \right)= \sigma(f\left( \boldsymbol{x} \right))$$

Predictions are obtained by computing the predictive distribution

$$\bar{\pi}_{*}\mathbb{\simeq E}\left[ \pi_{*} | X, \boldsymbol{y},\boldsymbol{x}_{*} \right]= \int\sigma\left( f_{*} \right)q\left( f_{*} | X, \boldsymbol{y},\boldsymbol{x}_{*} \right)df_{*}$$

where $\boldsymbol{x}_{*}$ denotes a new test point, $X$ denotes the training feature vectors, $\boldsymbol{y}$ denotes the training labels, and $f_{*}=f(\boldsymbol{x}_{*})$. This integral is numerically integrated.

All these information can be found in:

The source code of the Bayesian Logistic Regression model is available at: <https://github.com/mariamartinnez/BLR?tab=readme-ov-file>

For the GP classifier, we used the GPy library <https://gpy.readthedocs.io/en/deploy/>.

# References

1. Diederik P Kingma and Max Welling. Auto-encoding variational bayes. *arXiv preprint arXiv:1312.6114*, 2013.
2. Ignacio Peis, Pablo M Olmos, and Antonio Art´es-Rodr´ıguez. Unsupervised learning of global factors in deep generative models. *Pattern Recognition*, 134:109130, 2023.
